# Uncommon oral manifestations of dengue viral infection^☆^

**DOI:** 10.1016/j.bjorl.2016.10.001

**Published:** 2016-10-26

**Authors:** Carla Isabelly Rodrigues Fernandes, Luciano Elias da Cruz Perez, Danyel Elias da Cruz Perez

**Affiliations:** Universidade Federal de Pernambuco (UFPE), Departamento de Clínica e Odontologia Preventiva, Recife, PE, Brazil

## Introduction

Dengue infection is one of the most mosquito-borne viral diseases of humans worldwide that represents an important public health problem in over 100 tropical countries.^1^ Dengue virus infection may result in a broad spectrum of clinical presentations, ranging from asymptomatic or a mild, nonspecific fever, to classic dengue fever (DF), and severe presentations such as dengue hemorrhagic fever or dengue shock syndrome.^2^, ^3^

Most people infected with dengue viruses commonly develop dengue fever (DF), the signs and symptoms of which include high fever, myalgia, arthralgia, severe headache, retro orbital pain and maculopapular rash. Some patients sometimes develop nonspecific symptoms, such as nausea, vomiting, cough, dizziness, and diarrhea.^4^

In DF, immunosuppression is a common laboratory finding^5^ that represents one of the predisposing factors for oral pseudomembranous candidiasis. This fungal infection is characterized by a coating, or individual patches of pseudomembranous white slough that can be easily wiped away to reveal erythematous areas.^6^ Nevertheless, other oral findings rather than bleeding disorders have rarely been documented in DF patients.^2^ This report describes uncommon oral manifestations in a dengue fever patient, oral pseudomembranous candidiasis and transient lingual papillitis.

## Case report

**Figure 1:**
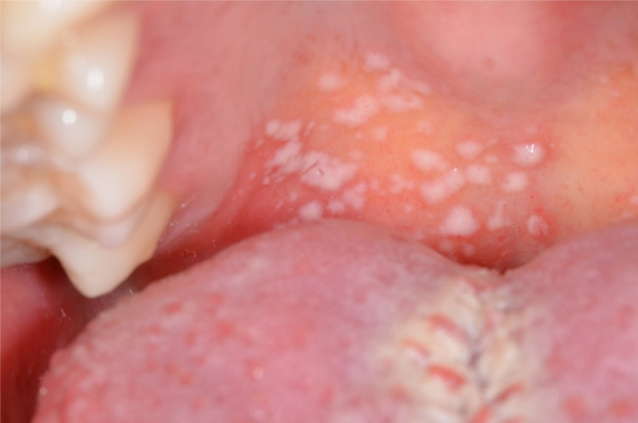
White pseudo-plaques on the soft palate.

**Figure 2:**
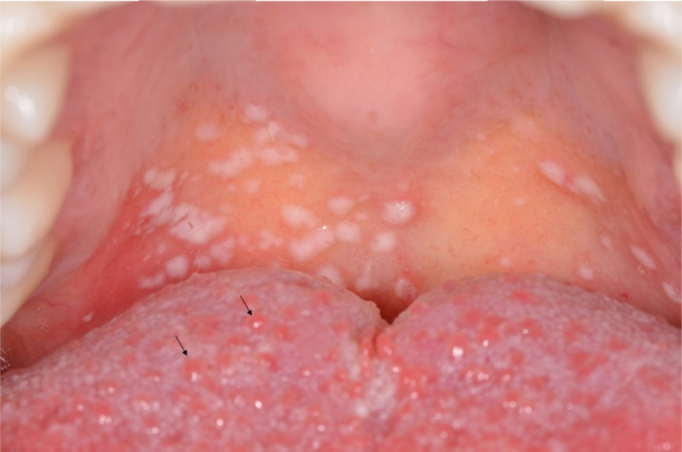
Hyperplasia of the fungiform papillae (arrows), with an edematous and erythematous appearance, characterizing a transient lingual papillitis.

The patient, 29 year-old woman was referred for evaluation of oral lesions with a 1 day history. In addition, the patient complained of severe pain when chewing. The patient was diagnosed with dengue fever and presented signs and symptoms associated with the disease, such as high fever, headache, muscle pain and rash. Serologic tests revealed the presence of immunoglobulins M (IgM) and G (IgG) for dengue antigens. Under medical prescription, the patient was taking acetaminophen (750 mg) four times a day and oral hydration. White cell count revealed 2400 cells/mm^3^; of these, 1248 mm^3^ were neutrophils. Her platelet count was 89,000 mm^3^. Intraoral examination revealed several white plaques located on the soft palate (Fig. 1). These plaques had a soft consistency and were removable by rasping, revealing an adjacent erythematous mucosa. No pain or other complaints were reported by the patient. The clinical diagnosis was pseudomembranous candidiasis. Furthermore, there was hyperplasia of the fungiform papillae that had an edematous and erythematous appearance, characterizing a transient lingual papillitis (Fig. 2). For oral candidiasis, the patient was treated with 5 mL of oral suspension of nystatin 100,000 UI four times a day for 1 week. After 3 days of treatment onset, the oral lesions presented significant improvement. No signs of the oral plaques were observed after 1 week. At the same time, the fungiform papillae were normal and the patient no longer complained of pain. Other systemic signs and symptoms associated with dengue fever had completely regressed.

## Discussion

The major specific and non-specific symptoms presented in dengue fever are well known and have been extensively described in the literature. However, the signs related to the oral cavity lack of further investigation and/or reporting. Oral mucosal involvement occurs in about 10% of the cases and is more commonly observed in patients with dengue hemorrhagic fever than with DF.^4^, ^7^ Oral manifestations of dengue infection have been described as gingival bleeding, and included by the World Health Organization as an unspecific finding of the disease. Despite this association, a case report from Brazil described the appearance of an enormous reddish swelling in the anterior upper gingiva and lip, as well as maculopapular lesions in the lower lip and left cheek mucosa, in addition to spontaneous gingival bleeding. Laboratory findings, including hematologic diagnosis and serologic tests, were consistent with the hypothesis of dengue infection.^2^ Tongue and soft palate may be also affected by bleeding disorders, such as erythema, petechiae and ecchymoses. Moreover, salivary gland disorder resulting in xerostomia have been also reported.^4^, ^8^, ^9^

As regards oral candidiasis, the literature reports that the use of large spectrum antibiotics, reduction of the patient immune capacity and xerostomia are the major risk factors for development of the condition. Immunosuppression is mostly associated with cases of leukemia and HIV.^6^ The association between DF and oral candidiasis has rarely been described in the literature, even as a non-specific sign. A study from South Asia investigated 104 children diagnosed with Dengue infection, who were admitted during a period of three months and the clinical findings showed that oral/oropharyngeal candidiasis was reported in 19 patients (18.2%). In 15 children, the infection affected the pharynx alone and in only four the entire oral mucosa was involved. None of them was previously immunosuppressed or taking steroids.^10^

In the present case, the presence of hyperplasia of the fungiform papillae was another peculiar oral sign observed. However, the reasons for this finding were not fully conclusive. The rise in volume of the fungiform papillae may be described as an inflammatory condition classified as Transient Lingual Papillitis (TLP) that has an abrupt onset, with one or more edematous, erythematous masses measuring 2–3 mm, as occurred in the present case. Affected papillae may become pustular, but even appear without pus. TLP is accompanied by moderate-to-severe pain and tenderness, also with considerable heat sensitivity,^11^, ^12^ similar to the present case. The multifactorial etiology of TLP include local unidentified infections, local irritation and stress.^11^ These facts suggest that the manifestation in the present case occurred due to the presence of a fungal infection in the oral cavity, in addition to emotional stress because of an infection with systemic symptoms, and one that could eventually progress to a serious medical event. Moreover, other authors have suspected that viral infections might also be included as a triggering factor for TLP.^12^, ^13^ In this case, the patient was primarily infected with the dengue virus. However, these possible associations require further investigation, since the etiology of TLP remains uncertain. To the best of our knowledge, this is the first report of TLP in a patient with DF.

## Conclusion

Given the importance of the oral manifestations in the present case, the thorough evaluation of oral findings in cases of dengue infection is extremely important, due to the rare reports presented in the literature. Therefore, particularly dentists and otorhinolaryngologists must be aware of different oral findings in order to provide a proper diagnosis and treatment of eventual oral candidiasis and/or other conditions.

## Conflicts of interest

The authors declare no conflicts of interest.
